# Substrates for Surface-Enhanced Raman Scattering Formed on Nanostructured Non-Metallic Materials: Preparation and Characterization

**DOI:** 10.3390/nano11010075

**Published:** 2020-12-31

**Authors:** Jan Krajczewski, Robert Ambroziak, Andrzej Kudelski

**Affiliations:** Faculty of Chemistry, University of Warsaw, Pasteur Str. 1, 02-093 Warsaw, Poland; jkrajczewski@chem.uw.edu.pl (J.K.); rambroziak@chem.uw.edu.pl (R.A.)

**Keywords:** SERS, surface-enhanced Raman spectroscopy, SERS substrates, multifunctional materials

## Abstract

The efficiency of the generation of Raman spectra by molecules adsorbed on some substrates (or placed at a very close distance to some substrates) may be many orders of magnitude larger than the efficiency of the generation of Raman spectra by molecules that are not adsorbed. This effect is called surface-enhanced Raman scattering (SERS). In the first SERS experiments, nanostructured plasmonic metals have been used as SERS-active materials. Later, other types of SERS-active materials have also been developed. In this review article, various SERS substrates formed on nanostructured non-metallic materials, including non-metallic nanostructured thin films or non-metallic nanoparticles covered by plasmonic metals and SERS-active nanomaterials that do not contain plasmonic metals, are described. Significant advances for many important applications of SERS spectroscopy of substrates based on nanostructured non-metallic materials allow us to predict a large increase in the significance of such nanomaterials in the near future. Some future perspectives on the application of SERS substrates utilizing nanostructured non-metallic materials are also presented.

## 1. Introduction

In the 1970s, it was observed that the intensity of Raman spectra generated by molecules adsorbed on some nanostructured surfaces is many orders of magnitude larger than the intensity of Raman spectra generated by the same number of molecules but not adsorbed. This effect was called surface-enhanced Raman scattering (SERS). In some cases, the generated SERS signal is so strong that it is possible to record a reliable SERS spectrum even of a single molecule [1,2]. This means that the SERS spectroscopy is one of the most sensitive analytical tools.

**Figure 1:**
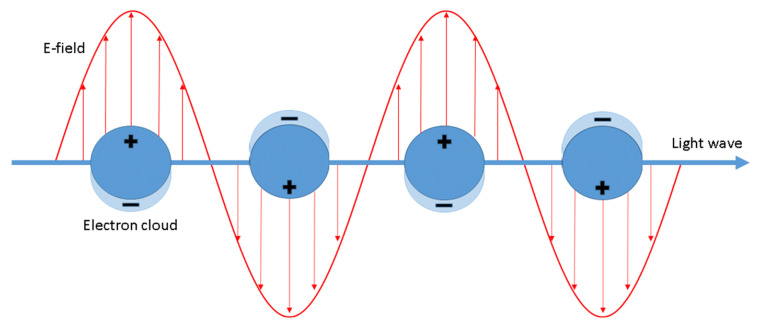
Schematic illustration of the surface plasmon resonance (SPR) phenomenon. Reproduced with permission [6]. Copyright, 2020, Springer Nature.

It is generally accepted that the enhancement of SERS spectra is due to the cooperation of two mechanisms: the electromagnetic and the chemical one. The electromagnetic mechanism is due to inducing the SERS substrate by the electromagnetic wave of the electric dipole (see Figure 1), which may lead to a significant local increase in the intensity of the electric field. The magnitude of the induced dipole (*p*) is proportional to [3]:

(1) $$p\sim \frac{\epsilon_{M}\left(\nu \right)-\epsilon_{out}\left(\nu \right)}{\epsilon_{M}\left(\nu \right)+2\epsilon_{out}\left(\nu \right)}$$

where: *ν* is the frequency of the excitation radiation, and *ϵ_M_*(*ν*) and *ϵ_out_*(*ν*) are the dielectric functions of the metal and the surrounding medium, respectively. When the denominator of the above fraction is close to zero, a strong electric dipole is induced, which leads to a very large local intensity of the electric field. This condition may be fulfilled in a significant part of the spectrum of visible radiation, for example for silver, gold, and copper nanoparticles. The SERS enhancement generated by this electromagnetic effect is roughly proportional to the fourth power of the field enhancement [4,5].

**Figure 2:**
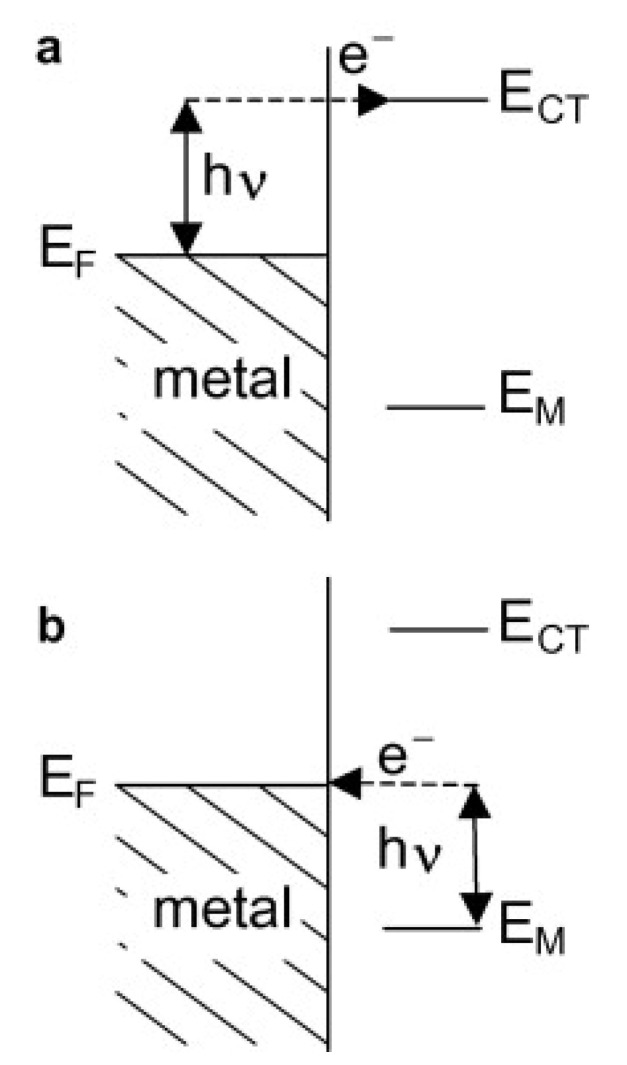
Schematic illustration of the energy levels involved in the charge transfer (CT) mechanism of the surface-enhanced Raman scattering (SERS) enhancement; hν—photon energy, E_F_—Fermi level energy, E_CT_—energies of occupied and unoccupied levels of the adsorbed molecules. (**a**) The electrons at the Fermi level of the metal are virtually excited into unoccupied molecular orbitals of the adsorbed molecule and back to the metal, (**b**) the electrons at the highest occupied molecular orbital can be virtually excited into the Fermi level of the metal and back to the adsorbed molecule. Reproduced with permission [7]. Copyright, 2009, Elsevier.

The second enhancement mechanism, which also leads to an increase in the efficiency of the generation of the Raman signal, is called the chemical enhancement. The interaction of adsorbed molecules with the metal substrate provides new electronic transitions for metal (or adsorbed molecule) electrons. The electrons at the Fermi level of the metal can be virtually excited into unoccupied molecular orbitals of the adsorbed molecule and back to the metal (see Figure 2a), or the electrons at the highest occupied molecular orbital can be virtually excited into the Fermi level of the metal and back to the adsorbed molecule (see Figure 2b). This means that a mechanism analogous for the standard resonance Raman process (that can generate a significant increase in the efficiency of the generation of the Raman signal) may be observed for adsorbed molecules [7].

To record the SERS spectrum, Raman scatterers (studied molecules) must be adsorbed at or be placed in a close proximity to the SERS-active material. The first surface-enhanced Raman spectra were published in 1974—in this case, the measurements were carried out on nanostructured silver [6]. During the next three decades, the SERS measurements were also carried out mainly on nanostructured plasmonic metals (Ag, Au and Cu), although other SERS substrates were also developed relatively quickly: in 1982, Loo recorded SERS spectra using as a SERS-active material polycrystalline TiO_2_ [8] and in 1983, Yamada and Yamamoto produced SERS-active materials by the deposition of some metals on the surface of NiO and TiO_2_ [9]. The number of SERS measurements carried out on composite substrates containing non-metallic nanostructured materials are still increasing. This is due to many significant advances of such SERS substrates in some applications of SERS spectroscopy, such composites usually have additional important functionalities that are not possible to obtain in standard SERS substrates produced only from plasmonic metals. In this review article, we present selected SERS substrates that utilize nanostructured non-metallic materials, including non-metallic nanostructured thin films or non-metallic nanoparticles covered by plasmonic metals and SERS-active nanomaterials that do not contain plasmonic metals. Some future perspectives of the application of composite SERS substrates are also discussed.

## 2. Non-Metallic Nanostructured Thin Films Covered by Plasmonic Metals

Nanostructured surfaces of semiconductors or insulators covered with plasmonic metals are very often used as SERS substrates. Few types of such substrates are even available commercially. In many cases, deposition of a plasmonic metal on the nanostructured surface of a semiconductor or an insulator may lead to SERS materials that generate higher and spatially more reproducible SERS enhancement factors than the standard SERS substrates produced from pure gold or silver. Moreover, the cost of the production of such substrates may be lower than the cost of manufacturing standard SERS substrates made from pure gold of silver. In this review, only substrates produced on selected inorganic substrates are described. In particular, we focused on the three most commonly used types of SERS substrates obtained from nanostructured films: TiO_2_, GaN and ZnO. Such SERS substrates have been extensively studied by many groups, and in this review, we want to show how complex these apparently simple systems are. SERS substrates may be, however, also produced on other nanostructured inorganic films, for example on ZrO_2_ [10]. Moreover, in addition to SERS materials formed on inorganic substrates, there are also widely used so called paper-based SERS substrates that are formed on cellulose [11,12,13]. This type of SERS substrate is also not described in this work.

### 2.1. Titanium(IV) Oxide

One of the most used materials with a nanostructured surface, on which a layer of a plasmonic metal is deposited to form SERS substrate, is an ordered film formed from titanium(IV) oxide nanotubes. Titanium can be quite easily covered by the layer formed from titanium(IV) oxide nanotubes by oxidative anodization. This method of synthesis of TiO_2_ nanotubes allows the obtention of nanostructures with different parameters depending on the electrolyte used [14] and the voltage [15] at which the reaction is carried out. The electrochemically obtained nanotubes are usually annealed for a few hours in order to change the structure of TiO_2_ from an amorphous one to an anatase or rutile structure [16]. Reconstruction of the crystallographic structure of TiO_2_ makes the layer of nanotubes on titanium substrate more stable and more mechanically durable.

**Figure 3:**
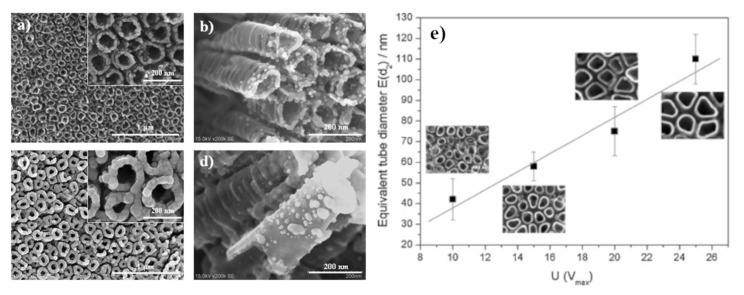
(**a**–**d**) SEM images of a layer of TiO_2_ nanotubes formed by the anodization of Ti foil in a glycol fluoride solution at 25 V covered by various amounts of Ag: (**a**,**b**) 0.01 mg·cm^−2^, (**c**,**d**) 0.06 mg·cm^−2^. (**e**) The diagram presenting the dependence between applied anodic voltage and the diameter of formed nanotubes. Reprinted with permission from [19]. Copyright 2010 Elsevier.

Pisarek et al. analysed properties of layers of plasmonic metals (Ag, Cu and Au) electrosputtered on previously obtained TiO_2_ nanotubes [17,18,19,20,21]. In these studies, TiO_2_ nanotubes were obtained by anodizing a thin titanium foil in an electrolyte containing 47.14% water, 52% glycerin and 0.86% NH_4_F *w*/*w*. The anodization potential significantly influenced the obtained nanotubes—the higher voltage, the larger the internal diameter of the formed nanotubes (see Figure 3) [19].

The anodization process lasted from about 13 min to 2 h. The anodization time affected the length of formed nanotubes but practically did not affect their diameter. In order to increase the stability of the formed material, the obtained nanotubes were annealed at a temperature of 600 °C for 1 h to 2 h, which led to the transition of the titanium(IV) oxide from amorphous to rutile face. When the annealing is carried out at lower temperatures (400 °C), instead of rutile, the anatase is obtained. In this case, the transition area between the Ti substrate and TiO_2_ nanotubes is, however, less developed, which leads to lower mechanical stability of the TiO_2_ layer [18]. The coating of the TiO_2_ nanotubes by the film of the plasmonic metal may be realized, for example, by the sputter-deposition technique. Table 1 shows how the amount (in mg·cm^−^^2^) of silver deposited by sputtering on the layer of TO_2_ nanotubes influences the structure of the formed film [19]. 

In addition to the dependence on the mass of deposited metal, the structure of formed plasmonic films depends on the kind of deposited metal (Ag, Au or Cu) and on the diameter of nanotubes on which the deposition is carried out. Although this dependence is relatively complex, some general rules on how the structure of formed film changes upon increasing the amount of the deposited metal may be, however, observed. When small amounts of sputtering metal are deposited, only single small nanoparticles on the surface of nanotubes are observed. Then, after deposition of an additional metal, we can observe the formation of metal rings on the end of nanotubes and the reduction in the diameter of nanotubes. After deposition of an additional portion of metal, the upper surface of the nanotubes becomes completely covered with a metal film. However, at all stages of metal deposition, semi-spherical nanoparticles with a diameter from 5 to 20 nm were visible. In the case of deposition of copper, the formation of the compact metal layer is observed at a significantly earlier stage of sputtering [21]. The formed films were investigated using Auger electron spectroscopy (AES). On the basis of the obtained AES results, it was found that the copper films deposited on the surface of nanotubes are covered with a thin layer of copper oxide. Formation of gold and silver oxides was not observed [21]. In addition, it was found that the MNN Auger signal from silver deposited on the TiO_2_ substrate was significantly wider than in the case of measurements of Auger spectra of pure silver. This suggests a significant interaction between the deposited silver nanostructures and the TiO_2_ substrate.

Interactions between deposited metal clusters and the TiO_2_ substrate do not only influence the structure of the deposited metal clusters. For example, sputtering copper on TiO_2_ nanotubes leads to a significant broadening of the Raman band at 900 cm^−^^1^ corresponding to the Ti=O stretching vibration [20]. Roguska et al. suggested that this broadening is due to the formation of mixed oxides during the sputtering of Cu onto the TiO_2_ substrate (titanium oxide is mixed with copper ions).

Mao et al. observed that the SERS substrates produced by the deposition of silver on TiO_2_ nanotubes can self-clean when illuminated with UV radiation [22]. In this case, TiO_2_ nanotubes were obtained by 15 min anodization of titanium in a glycol solution of HF (0.5% vol) at various voltages (between 20 V and 60 V). Originally formed nanotubes from amorphous TiO_2_ were heated at 500 °C for 2 h in order to change the structure of TiO_2_ to anatase. Then, a thin layer of silver (0.03 mg/cm^2^) was deposited. Mao et al. found that when such systems are stored in humid air, their illumination with UV radiation (with the wavelength of 365 nm) leads to changes in the structure of the deposited silver clusters [22]. One of the effects induced by the UV irradiation is the transfer of silver to the inner parts of the nanotubes. As mentioned above, Mao et al. also noticed that after UV irradiation, the Raman signal from the previously adsorbed organic compound significantly decreases, which means that this substrate has self-cleaning properties.

Silver nanostructures may be deposited on the surface of TiO_2_ nanotubes not only by the sputtering in vacuum, but also by a chemical reduction of a silver salt, for example, through the polyol process [23]. Meng and Xie found that when silver clusters are deposited using the polyol process, only a few silver nanoparticles were attached to the walls of TiO_2_ nanotubes, most formed nanoparticles were deposited on the top edges of nanotubes [23]. In the next step, Meng and Xie decided to cover formed TiO_2_/Ag composite with a layer of graphene oxide (GO). It has been shown that the presence of GO facilitates adsorption of methyl blue on the surface of such material. This is due to the π–π interactions of graphene rings with the rings of methyl blue and due to a significantly developed surface of such composite. The obtained TiO_2_/Ag/GO composite was tested as a SERS substrate. Meng and Xie found that the intensity of the SERS spectra of methyl blue adsorbed on TiO_2_/Ag/GO composite is larger than in the case of SERS measurements on the TiO_2_/Ag composite before covering with GO [23]. The authors believe that the GO layer is so thin that it does not influence the plasmonic interactions, and the aforementioned better adsorption increases the concentration of methyl blue on the surface. After 60 days storage of SERS substrate covered with GO, the decrease in the intensity of the SERS spectrum of methyl blue is only 5%, which is much smaller than in the case of the sample without GO, where the decrease in the intensity of the measured SERS signal is 55%. The authors believe that graphene oxide protects silver nanoparticles from oxidation. Meng and Xie also found that the covering with GO improves the efficiency of self-cleaning of such SERS substrate under the influence of UV radiation. They associate this effect with the possibility of electron transfer and a larger surface area of the GO layer. 

It is also possible to attach previously synthesized plasmonic nanoparticles to TiO_2_ nanotubes [24,25]. In such a case, one can attach a large number of very similar (having practically the same shape and size) plasmonic nanoparticles to the TiO_2_ nanotubes. For example, Ambroziak et al. immobilized previously synthesized cubic silver nanoparticles on TiO_2_ nanotubes [25]. Cubic silver nanoparticles have been synthesized using the polyol method developed by Skrabalak et al. [26]. When illuminated by the proper electromagnetic wave, silver cubic nanoparticles generate at their corners and edges a significantly enhanced electric field [27], and hence, the enhancement of the SERS spectrum generated by such plasmonic nanostructures is significantly larger than that generated by the respective spherical plasmonic nanoobjects. Ambroziak et al. found that the SERS enhancement generated by the SERS substrate produced by the attachment of cubic silver nanoparticles to TiO_2_ nanotubes (when only a part of the outside surface is covered by the plasmonic material) is even higher than the SERS enhancement generated by the standard electrochemically nanostructured solid silver substrate. Moreover, the spatial reproducibility of the SERS enhancement factor for such composite TiO_2_-nanotubes/cubic-Ag nanoparticles substrate is larger than that for the standard electrochemically nanostructured silver substrate [24].

Sun et al. analysed how the wall thickness of TiO_2_ nanotubes, on which Ag nanoparticles were deposited, influences the SERS enhancement factor achievable on this material [28]. In other words, the purpose of these studies was to find the wall thickness of TiO_2_ nanotubes, which allows us to maximize the number of *hot spots* (places where especially large enhancement of the electric field is generated—*hot spots* are observed in narrow slits between plasmonic nanostructures or on sharp structures on surfaces of plasmonic objects) created during the silver deposition by e-beam evaporation, and hence, allows the obtention of the SERS substrate generating the highest SERS enhancement factor. Sun et al. found that the optimal wall thickness of TiO_2_ nanotubes is about 20 nm. The SERS enhancement factor for 2-mercaptobenzoxazole adsorbed on such SERS substrate (with the wall thickness of TiO_2_ nanotubes equal to 20 nm) is 2.26 × 10^8^. As for other described above TiO_2_-nanotubes/Ag composites, the obtained SERS substrate is self-cleaning when illuminated with UV radiation. Sun et al. carried out the following experiment three times: 2-mercaptobenzoxazole was adsorbed on the TiO_2_-nanotubes/Ag composite, the SERS spectrum was measured, then the substrate was irradiated for 20 min using a UV lamp and was rinsed with water. The spectrum of the “cleaned substrate” was measured again. It was found that after UV irradiation, the SERS spectrum of 2-mercaptobenzoxazole disappeared, and after reapplying this compound to the SERS substrate, practically identical SERS spectra were obtained.

### 2.2. Gallium Nitride

Another often used surface-nanostructured material, on which a layer of a plasmonic metal can be deposited to form an efficient SERS substrate, is porous gallium nitride. In 2005, Bohn et al. published a paper showing that the surface-nanostructured GaN coated with gold and silver is very efficient SERS substrate [29]. After the cleaning process (in principle successive immersion in the following solvents: acetone, 2-propanol, 65% HNO_3_, water and methanol), GaN substrates were placed in an etching solution of CH_3_OH, HF (49%) and H_2_O_2_ (30%) in 1:2:2 volume ratio and illuminated for 90 min with UV radiation. This made the surface of the GaN sample very porous. Subsequently, the etched surface of GaN was cleaned by washing with water and methanol and then sonicated for 15 min to remove ridge structures. The resulting porous structure formed on the etched surface of GaN resembles nanotubes. Then, a layer of gold and silver was deposited. Deposition of the layer of plasmonic metal can be realized by the vacuum evaporation or by the chemical reduction of a silver or gold compound. In the case of the chemical coating, before the deposition of the plasmonic film, the nanostructured GaN substrates were immersed in concentrated HF to remove surface oxides. Plating with gold was performed using a three-step method containing: sensitization, activation, and platting. The sensitization was performed by placing the etched GaN substrate in water–methanol diluted solution of SnCl_2_ and CF_3_COOH. Activation was performed by the immersion of the GaN substrate in a mixture of a solution of AgNO_3_ and aqua ammonia. The plating step was performed by the immersion of the GaN substrate in a mixture of diluted commercially available Au plating solution and NaHCO_3_ and Na_2_SO_3_. Then, the formaldehyde was added as a reducing agent. After the deposition of the gold film, the substrates were rinsed with water and with 25% HNO_3_. The silver films were obtained by a similar method; the only difference was that in this case the plating solution was identical to the activation solution (see above)—immediately prior to silver deposition, formaldehyde was added to the plating solution to act as a reducing agent. Gold and silver films deposited using the described above chemical methods were composed mainly from many spheroidal nanostructures. Silver and gold films have been also deposited on the nanostructured GaN substrates by thermal evaporation. In this case, formed films were significantly flatter. The SERS enhancement factors generated by the formed SERS substrates were determined using malachite green isothiocyanate as a Raman scatterer. In the case of nanostructured GaN substrates covered with the gold layer, the SERS enhancement factor determined for the sample with the chemically deposited layer was equal to 2 × 10^4^, whereas for the sample with the evaporated gold layer the SERS enhancement factor was equal to 2 × 10^5^. In the case of samples with a silver film, a larger SERS enhancement factor (equal to 1 × 10^8^) was obtained on the substrate covered using the chemical process than on the substrate covered by the vacuum evaporation (5 × 10^7^) [29].

**Figure 4:**
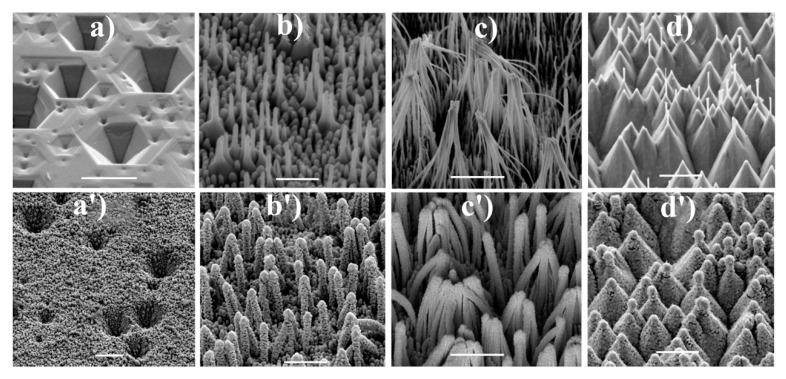
SEM images of GaN samples after: (**a**) orthodox etching at 420 °C for 10 min, (**b**,**c**) galvanic photo-etching for 8 and 12 min, respectively, (**d**) electroless photoetching for 120 min. (**a’**–**d’**) shows SEM images of similar samples after sputtering of an Au-Ag alloy. The samples are tilted 45°. The scale bars represent 1 μm. Reprinted with permission from [37]. Copyright 2018 Elsevier.

Many materials for SERS measurements based on nanostructured GaN substrates containing the surface nanopillars shaped-like conical sheaves have been produced by Weyher et al. (example morphologies of studied materials are shown in Figure 4). On such surface-nanostructured GaN substrates, gold [30,31,32,33], gold and silver alloy [34] or gold and copper alloy [35] were deposited. In this case, the nanostructuring of GaN surface was realized by pre-etching in a 10% HF solution and subsequent photo-etching using a 300 W xenon UV lamp in a solution of 0.02 M KOH and 0.02 M K_2_S_2_O_8_. Then, pure plasmonic metals or their alloys were electrosputtered onto nanostructured GaN substrate. The obtained morphology of the nanostructured GaN surface allows the obtention of SERS substrates with a significant number of *hot spots*. For example, after the deposition of the gold layer with a thickness of 90 nm [32], the SERS enhancement factor for the obtained SERS substrate was determined as equal to 2 × 10^6^ (as a Raman scatterer p-mercaptobenzoic acid was used). The SERS spectrum measured using this substrate has very high reproducibility—relative standard deviation of the intensity of the spectra measured at various sites was only 10%. Produced GaN/Au SERS substrate was also very stable over time, after 10 months, the intensity of the measured SERS spectrum decreased by only 10% compared to the freshly made substrate. Weyher et al. also studied the influence of the annealing at various temperatures of the GaN/Au substrates on their structure and their SERS activity [36]. Annealing up to the temperature of 200 °C increases the SERS enhancement factor, which is attributed to the generation of more *hot spots*. Annealing in the temperatures between 250 and 350 °C decreases the intensity of the measured SERS spectrum due to the coalescence of nanoparticles. Increase in the intensity of the measured SERS spectrum is observed again for GaN/Au substrates annealed in the temperatures between 450 and 750 °C, which is associated with the formation of crystallographic planes enabling surface binding sites and forming SERS *hot spots*.

As mentioned above, nanostructured GaN substrates with nanopillars shaped-like conical sheaves on the surface have been also covered with some alloys of gold. Using gold alloys instead of the pure gold allows the modification of the plasmonic properties of the deposited film and also allows to easily increase the roughness of the film by dealloying. For example, GaN substrates covered with Au:Ag alloy (initially 70/30 wt%) after soaking for one day in nitric(V) acid become (due to the dealloying the content of silver drops to 19%) significantly more rough and more SERS-active. The SERS enhancement factor generated by this substrate in measurements with p-mercaptobenzoic acid was estimated as equal to 1.5 × 10^7^ [34]. The dealloyed Au/Ag film is chemically very resistant, for example, the content of silver does not change significantly even after further long soaking in nitric(V) acid. This means that the obtained substrate combines the high chemical stability of gold and the significantly better plasmonic properties of silver. In another experiment, such type of nanostructured GaN substrates have been covered by AuCu alloy (40/60 at%) [34]. Again, after dealloying in nitric acid, the Cu content drops to 10% and the SERS enhancement factor achieves the value of 2 × 10^5^.

**Figure 5:**
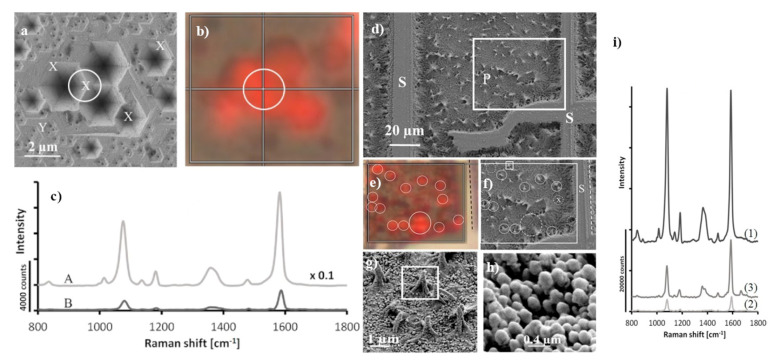
(**a**,**b**) SEM images and corresponding SERS intensity map of the spectrum of p–mercaptobenzoic acid adsorbed on the surface of GaN/AuAg (plot represents intensity of the band at 1590 cm^−^^1^), (**c**) recorded SERS spectra of p–mercaptobenzoic from A: point indicated by white circle on (**a**) and B: point indicated as Y. (**d**,**f**–**h**) SEM images of photo-etched GaN samples, (**e**) SERS intensity map of the band at 1590 cm^−^^1^ of p–mercaptobenzoic acid and (**i**) SERS spectra of p–mercaptobenzoic acid recorded on substrates having various GAN morphologies: 1 nano-pillars, 2 on the matrix, 3 at the position matched as X in (**a**). Reprinted with permission from [37]. Copyright 2018 Elsevier.

The surface of GaN can be also nanostructured in another manner. For example, Weyher et al. at first etched the surface of GaN in a molten eutectic KOH—NaOH mixture, which led to the creation of inverted pyramids on the surface of the GaN substrate, and then the previously described process of the creation of nanopillars shaped-like conical sheaves was realized [35,37]. In the next steps, AuCu alloy (40/60 at%) [35] or AuAg alloy (70/30 at%) [37] were deposited and the obtained films were further etched in nitric acid. Weyher et al. found that the initial nanostructuring leading to the creation of the inverted pyramids induced an additional increase in the SERS enhancement factor, for example, in the case of experiments with AgCu alloy, to 5 × 10^5^ (as mentioned above, the SERS substrate produced without the step of the creation of the inverted pyramids on the GaN surface generates the SERS enhancement factor equal to 2 × 10^5^ [35]. Figure 5 shows example SERS-activity maps for various GaN samples covered with AuAg alloy [37].

**Figure 6:**
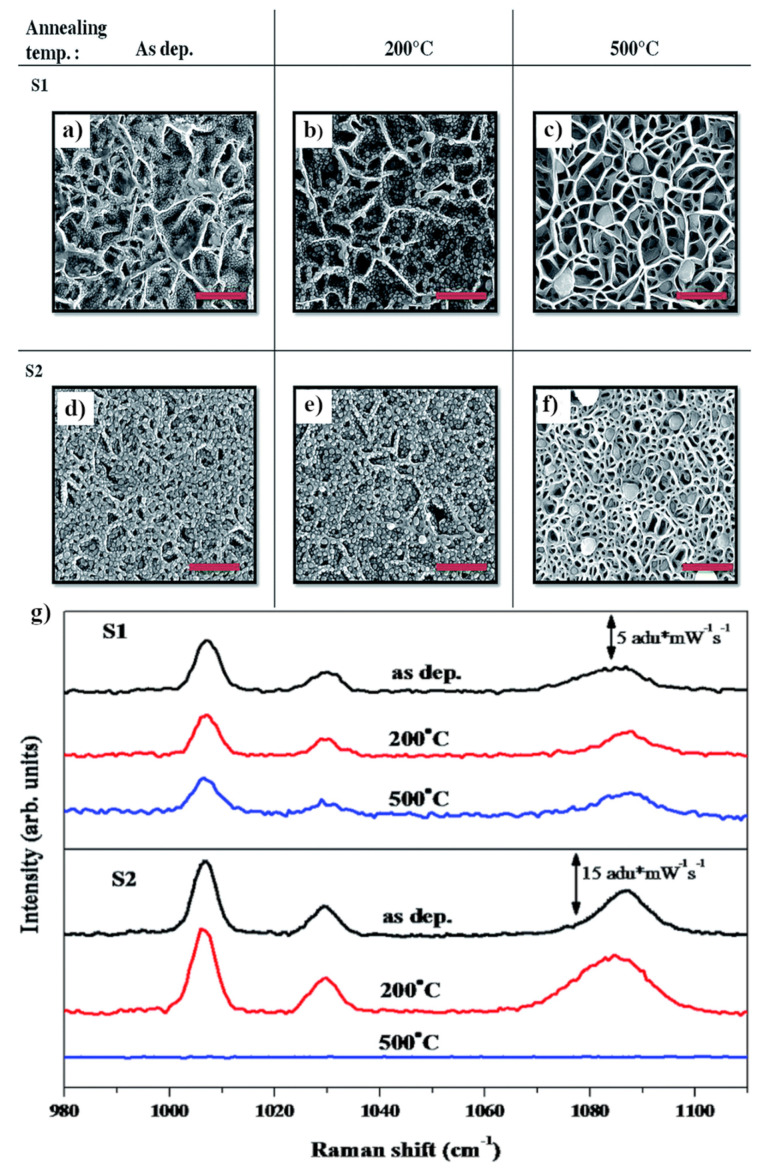
SEM images of GaN nanowalls covered with silver: less densely packed (S1) and more densely packed (S2). (**a**,**d**) Without annealing, (**b**,**e**) after annealing at 200 °C, (**c**,**f**) after annealing at 500 °C. (**g**) SERS spectra for substrates S1 and S2. Thiophenol was used as a test molecule. Reprinted with permission from [38]. Copyright 2015 Royal Society of Chemistry.

An interesting method of preparation of nanostructured GaN films has been proposed by Shivaprasad et al. [38]. This group prepared a sponge-like GaN network through the plasma assisted molecular beam epitaxy. The change of the parameters of the epitaxial growth allows the preparation of various sponge-like GaN networks, for example, with the average wall thickness of 40 nm or much more densely packed structures with the wall thickness of 60 nm (see Figure 6). Then, on such nanostructured GaN substrates, silver was deposited using electron beam evaporation. The SERS enhancement factor after deposition of the amount of silver required to form the silver film with the average thickness of 13 nm was estimated as equal, on average, to about 10^5^—differently prepared substrates generate slightly different SERS enhancement factors. In general, higher SERS enhancement factors were observed on more densely packed GaN nanostructures, which is probably related to the larger number of *hot spots* created in this system.

### 2.3. Zinc Oxide

Efficient SERS substrates are also often produced utilizing the nanostructured film of zinc oxide. For example, in 2010, Ozaki et al. synthesized a very efficient SERS substrate by the deposition of a thin layer of silver on the film of zinc oxide nanorods [39]. Zinc oxide nanorods with hexagonal arrangement were obtained by hydrothermal synthesis. At first, a zinc foil cleaned in water and alcohol was placed in an autoclave filled with an aqueous solution of 0.9 M NaCl and 5.56 mM acetic acid. The temperature was raised to 120 °C and the zinc foil was kept for 14 h at this temperature. This led to the formation of ZnO nanowires on the surface of the foil, with a diameter between 200 and 500 nm and the length of about 2 μm. Then, the deposition of silver nanostructures was carried out by the immersion of the Zn/ZnO sample in AgNO_3_ solution. Ozaki et al. found that the Ag nanostructures were only formed at the top of the nanorods, which is probably caused by the mechanism of the transfer of the electric charge from the Zn substrate only along the ZnO nanorods. Changing the time of silver deposition and the concentration of the AgNO_3_ solution, it is possible to control the size and density of the formed Ag nanoparticles. For example, when the ZnO substrate was immersed in 10^−^^5^ M AgNO_3_ solution for 1 min, only a few small nanoparticles with a size between 10 and 20 nm are formed at the top of ZnO nanorods. With the increase in the concentration of AgNO_3_ solution, larger Ag nanoparticles are formed. Ozaki et al. found that the highest SERS enhancement factors (ca.·10^6^) were measured using the substrates obtained by the immersion of ZnO substrates for 1 min in a AgNO_3_ solution with the concentration in the range between 10^−^^4^ and 10^−^^3^ M [39]. 

**Figure 7:**
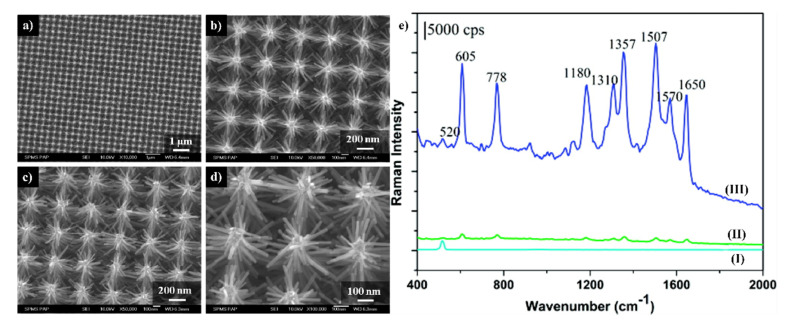
(**a**–**d**) SEM images of the ordered Si/ZnO nanotrees: (**a**,**b**) top view, (**c**,**d**) 20^o^ titled view, (**e**) SERS spectra of molecules of rhodamine 6G adsorbed from the solution with the concentration of 10^−^^6^ M collected from: (I) Si/ZnO nanotrees, (II) Ag nanoparticles substrate prepared by the sputtering method, (III) Si/ZnO nanotrees decorated by Ag nanoparticles. Reprinted with permission from [40]. Copyright 2010 American Chemical Society.

An interesting SERS substrate containing nanostructured ZnO was developed by Fan et al. [40]. At first, a nanopillar Si array was achieved using standard top-down photolithography pattering followed by dry etching and wet etching to remove metal and oxides. The obtained Si nanopillars were 300 nm high, 150 nm in diameter and the distance between them was about 400 nm. ZnO seeds needed for the branch growth were obtained via the atomic layer deposition using diethyl zinc and water. Then, the prepared substrate was putted into Zn(NO_3_)_2_ and hexmethylenetetramine solution and kept at 95 °C for 3 h. This process led to the formation of ZnO branches that were perpendicularly directed to the Si nanopillars, were 150–200 nm long and about 30 nm in diameter (see Figure 7). Finally, the silver was deposited by the photochemical method: the substrate was placed in a 0.1 M AgNO_3_ solution for 20 min and then was illuminated for 3 min using a 25 W UV lamp. The silver nanoparticles formed on the ZnO branches had a diameter of about 20–30 nm. Fan et al. determined the SERS enhancement factor for this system as equal to 10^6^ [40].

**Figure 8:**
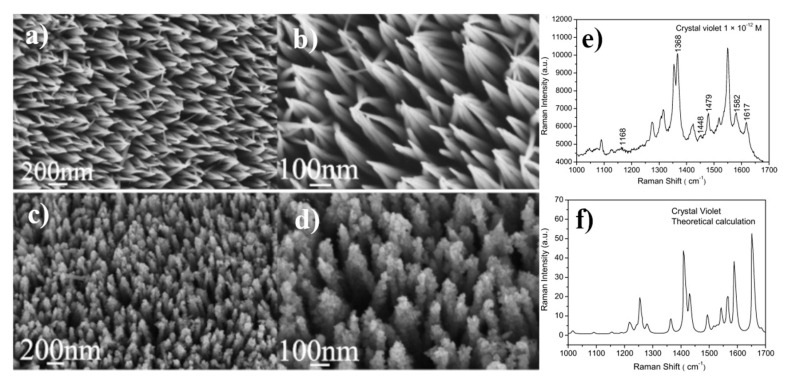
(**a**,**b**) SEM images of ZnO nanoarrays; (**c**,**d**) SEM images of ZnO/Ag composite nanoarrays, (**e**) SERS spectrum of crystal violet adsorbed on ZnO/Ag composite nanoarrays from 10^−^^12^ M crystal violet solution, (**f**) theoretically calculated Raman spectrum of crystal violet. Reprinted with permission from [41]. Copyright 2011 Elsevier.

Additionally, ZnO nanoneedles covered with silver nanoparticles can be used as SERS substrates [41]. ZnO nanoneedles were formed by the immersion for 12 h of a cleaned zinc foil in a 0.5 M Zn(NO_3_)_2_ and 3 M KOH solution. The sample prepared in this way was rinsed with water and dried. Obtained ZnO nanoneedles were perpendicularly oriented to the metal substrate and have a diameter of 6–10 nm. Silver nanostructures on a ZnO substrate were obtained by the immersion of the nanostructured ZnO substrate in 10^−^^3^ M AgNO_3_ solution and exposing it to the sun radiation for 20 min. This leads to the formation of monocrystalline silver nanoparticles with a diameter of 10 nm, which were attached to the surface and tips of ZnO nanoneedles (see Figure 8). Wang et al. showed that such SERS substrates may be used to detect Sudan II and Sudan IV dyes at concentrations as low as 10^−^^12^ M [41].

**Figure 9:**
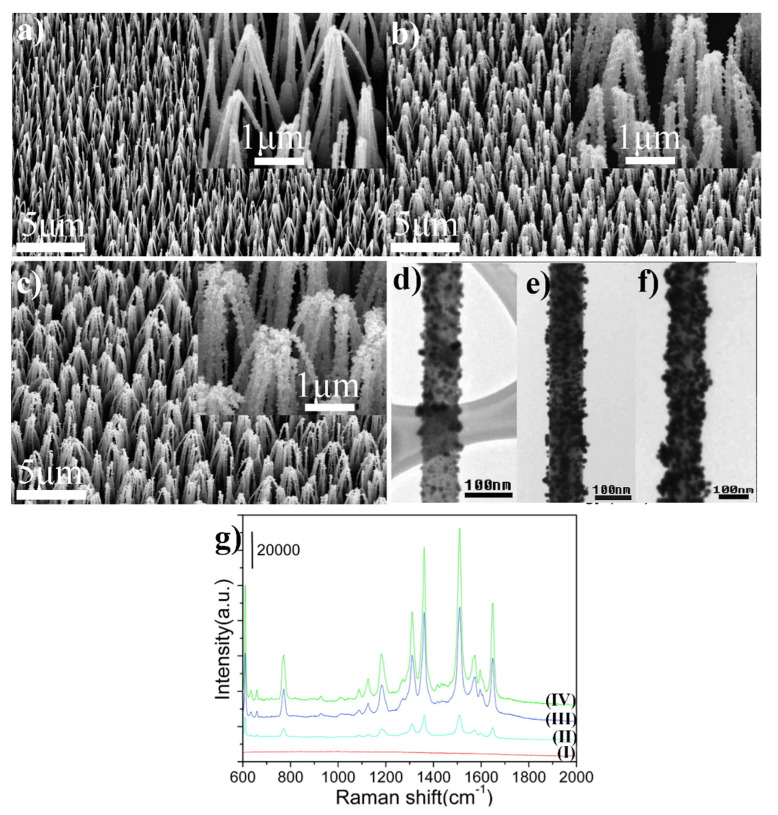
(**a**–**c**) SEM images of ZnO/Au nanoneedle arrays prepared at different HAuCl_4_ concentrations: (**a**) 0.1, (**b**) 0.3 and (**c**) 0.5 mM. Inset shows corresponding high magnification SEM images. (**d**–**f**) TEM images of ZnO/Au nanoneedles prepared at different HAuCl_4_ concentrations: (**d**) 0.1, (**e**) 0.3 and (**f**) 0.5 mM. (**g**) SERS spectra of molecules of rhodamine 6G adsorbed on: (I) ZnO (II), (III) and (IV) ZnO/Au produced using HAuCl_4_ at various concentrations: 0.1, 0.3 and 0.5 mM, respectively. Reprinted with permission from [42]. Copyright 2009 American Chemical Society.

Highly SERS-active material may be also obtained by the deposition of gold nanoparticles on the nanostructured ZnO. For example, Lee et al. produced SERS substrates by the deposition of gold nanoparticles on ZnO nanorods and nanoneedles (see Figure 9) [42]. ZnO nanoneedle and nanorod arrays were grown on an aluminium-doped zinc oxide by chemical vapor deposition. Then, the nanostructured ZnO substrates were immersed in water–methanol solution of HAuCl_4_ (the concentration of Au ions was in the range between 0.1 and 0.5 mM), the sample was placed in an autoclave and kept at 120 °C for an hour. The amount of the deposited gold on the surface of ZnO nanoneedles can be controlled by altering the concentration of HAuCl_4_. When the amount of the deposited gold is high, ZnO/Au nanoneedles form bundles. The obtained substrates were tested for the enhancement of the Raman spectrum using rhodamine 6G. The highest achieved enhancement factor was 1.2 × 10^7^ [42].

Alessandri et al. showed that the SERS substrates produced by the sputtering of gold on ZnO nanorods have self-cleaning properties [43]. In order to obtain the ZnO nanorods, at first, ZnO islands on Si wafers were formed by atomic layer deposition. The production of ZnO islands on Si wafers allows the control of the distribution of sites of future ZnO growth. Then, the growth of ZnO nanorods was carried out by the hydrothermal method, in short: the Si wafer with ZnO seeds was placed in an aqueous solution of 0.02 M Zn(NO_3_)_2_ and 0.04 M hexamethylenetetramine and was kept at 90 °C for 6 h. The produced nanorods were between 300 and 400 nm in length and their diameter was about 30–40 nm. On the obtained ZnO nanorods, gold was sputter deposited at room temperature. The obtained material was tested as a substrate for SERS measurements: it was found that it is possible to record the SERS spectrum of methylene blue even at the concentration of 10^−^^12^ M. Additionally, the self-cleaning properties of the substrate have been demonstrated. After the recording of the SERS spectrum of methylene blue adsorbed from the solution with a concentration of 10^−^^4^ M, the substrate was irradiated with a UV lamp. It was shown that after 15 min of irradiation, the SERS signal became very weak, and after 30 min of exposure, the characteristic SERS spectrum of methylene blue completely disappeared.

## 3. Non-Metallic Nanoparticles Covered by Plasmonic Metals

To produce effective SERS substrates, plasmonic metals are deposited not only on the macroscopic nanostructured non-metallic materials, but sometimes plasmonic metals are also attached to some non-metallic nanoparticles. Although SERS substrates formed on non-metallic nanoparticles are not used as often as materials for SERS measurements formed on macroscopic nanostructured materials (some types of macroscopic SERS substrates, for example, formed on nanostructured macroscopic GaN are even commercially available), there are also many important types of SERS substrates that have the form of such nanocomposites. In this section, we describe three selected examples of the most important substrates produced by combining metallic and non-metallic nanostructures that have some additional functionalities in comparison to the SERS substrates formed only from plasmonic metals. 

### 3.1. Iron Oxides

Probably the most important type of SERS materials obtained from the nanostructured non-metallic nanoparticles and the plasmonic metals are magnetic SERS substrates. Introducing the additional functionality (magnetism) to the SERS substrates significantly facilitates the deposition of such materials and their removal after the measurement—these processes may be easily carried out with an inexpensive commercially available neodymium magnet. For example, very often plasmonic nanoparticles used as SERS substrates are deposited on an analysed surface by the deposition of a drop of their sol and evaporation of the solvent. Unfortunately, when this simple method of deposition is used, due to the so-called coffee ring effect, a significant accumulation of nanoparticles at the boundary of the area covered by the sol is usually observed. When the deposited nanostructures also have magnetic properties, and the deposition is carried out in the magnetic field (for example, generated by a strong magnet), the coffee ring effect is usually eliminated, and homogeneously distributed films of nanostructures are formed. SERS magnetic–plasmonic substrates may be also relatively easy removed from the analysed sample using a strong magnet. Therefore, many groups are working on the development of SERS substrates with magnetic properties. For example, Sun et al. synthesized Fe_3_O_4_@AuAg alloy core–shell nanoparticles [44]. At first, Fe_3_O_4_ nanoparticles were synthesized by solvothermal reaction; briefly: a solution of FeCl_3_·6H_2_O, sodium citrate and sodium acetate in ethylene glycol was heated at 200 °C for 10 h. Then, Fe_3_O_4_ nanoparticles were functionalized with NH_2_-terminated silane coupling reagent (3-aminopropyltrimethoxysilane), the sol of functionalised Fe_3_O_4_ nanoparticles was mixed with a sol of small Au nanoparticles and the obtained mixture was sonicated, which led to the formation of Fe_3_O_4_-Au composites. In the second stage, the obtained Fe_3_O_4_-Au composites were kept in an Au-Ag alloy shell growth solution (which contained: HAuCl_4_, AgNO_3_, K_2_CO_3_, NH_3_, and HCHO). By changing the amount of HAuCl_4_ and AgNO_3_, the Fe_3_O_4_@AuAg alloy nanoparticles with different component ratios of Au and Ag were prepared. Changing the molar fraction of Ag and Au led to a significant change in the plasmon properties of the obtained structures.

As mentioned above, many other types of magnetic SERS materials have been synthesized, for example: agglomerates of gold and carbon-covered Fe_3_O_4_ nanoparticles [45], silver nanoflowers formed on the surface of core–shell Fe_3_O_4_@Au nanoparticles [46] or even four-layer Fe_3_O_4_@Ag@SiO_2_@Au microspheres [47]. Attaching a plasmonic nanostructure to the magnetic part of the composite need not cause a significant reduction in the SERS activity of the obtained SERS substrate, for example, the SERS enhancement factor achievable on silver nanoflowers formed on the surface of core–shell Fe_3_O_4_@Au nanoparticles was estimated to be equal to 2.2 × 10^9^ [46].

An interesting universal method of producing of magnetic SERS substrates has been proposed by Kolataj et al. [48]. This group found that various metallic nanoparticles easily bond to the surface of magnetic γ–Fe_2_O_3_ nanoparticles functionalized with (3-aminopropyl)trimethoxysilane. Kolataj et al. showed that when this method of formation of agglomerates is used, the initial size and shape of the noble metal nanoparticles do not change during the process of linking; therefore, highly anisotropic plasmonic structures may be attached to the magnetic nanoparticles. Kolataj et al. also emphasized that such composites form homogeneous layers in the magnetic field, so reproducible SERS substrates may be easily formed using this material [48]. 

### 3.2. Molybdenum Oxides

The other important type of SERS materials obtained from the nanostructured non-metallic nanoparticles and the plasmonic metals are nanoparticles of molybdenum oxide on which a plasmonic metal has been deposited. These type of SERS substrates are very promising because, as described in detail in the next section, nanostructured molybdenum oxides (both MoO_2_ and MoO_3_) are very active in SERS spectroscopy, and the SERS enhancement factor generated by nanostructured MoO_2_, MoO_3__−__x_ or MoO_3_ may even reach the values generated by standard silver and gold SERS substrates. Combining molybdenum oxide and metallic plasmonic nanostructures gives SERS substrates for which both electromagnetic and chemical enhancements are very large—so an interesting synergistic enhancement effect is observed [49]. An example of such a substrate was formed by Shi et al., who synthesized MoO_3_@Ag hybrid nanostructures. This group separately synthesized MoO_3_ nanowires (using the hydrothermal method, briefly: aqueous solution of ammonium molybdate and nitric acid was heated at 180 °C for 12 h using a Teflon-lined stainless autoclave), silver nanoparticles and then self-assembly of Ag nanoparticles on the surface of MoO_3_ nanowires was carried out using (3-aminopropyl)diethoxy methylsilane as a coupling agent [49]. 

Effective combination of the electromagnetic and chemical SERS enhancements has been also reported for nanostructured molybdenum oxides decorated with gold nanoparticles [50]. Although, as mentioned above, nanostructured molybdenum oxides are already an effective SERS substrate, the deposition of gold nanoparticles leads to a further increase in the SERS enhancement factor generated by this material (in this case, by a factor of eight).

### 3.3. Titanium(IV) Oxide

**Figure 10:**
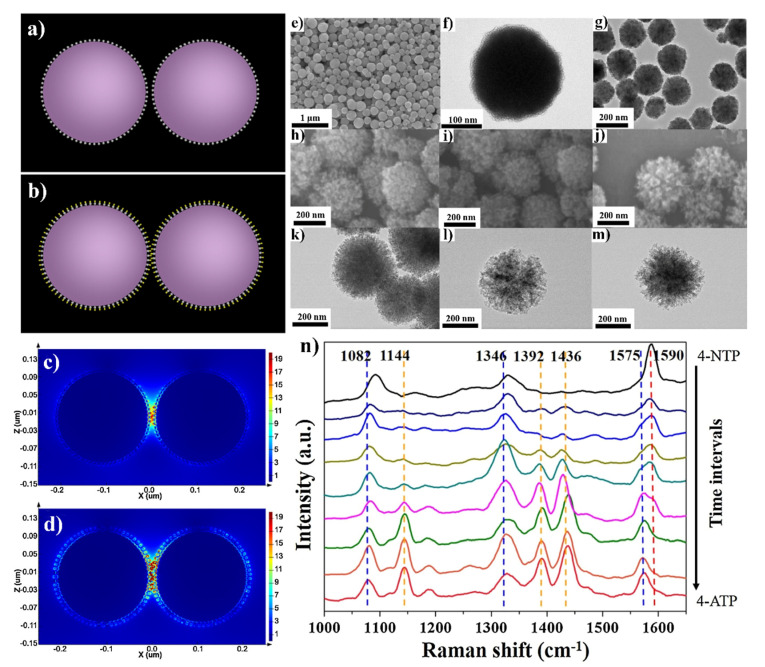
(**a**) The simulation model of Fe_3_O_4_@TiO_2_@Ag and (**b**) Fe_3_O_4_@TiO_2_@AuAg nanoparticles, (**c**) the calculated electromagnetic field distribution around Fe_3_O_4_@TiO_2_@Ag and (**d**) Fe_3_O_4_@TiO_2_@AuAg nanoparticles. The incident light wavelength was 785 nm. (**e**) SEM images of Fe_3_O_4_ spheres, (**f**) TEM image of Fe_3_O_4_@TiO_2_ core–shell sphere, (**g**) SEM images of Fe_3_O_4_@TiO_2_@Ag sphere, (**h**–**j**) SEM images of Fe_3_O_4_@TiO_2_@AuAg nanospheres with various molar ratio of Ag:Au: (**h**) 1:2, (**i**) 1:1, (**j**) 2:1, (**k**–**m**) the corresponding TEM images, (**n**) SERS spectra illustrating the reduction of 4-nitrophenol to 4-aminophenol catalysed by Fe_3_O_4_@TiO_2_@Au-Ag nanospheres. Reprinted with permission from [51]. Copyright 2016 Elsevier.

An interesting functionality that can be achieved by SERS substrates due to the attachment of another material to the plasmonic nanoparticles is self-cleaning under UV irradiation. For example, Shen et al. formed multifunctional Fe_3_O_4_@TiO_2_@Ag-Au microspheres (see Figure 10), which were efficient SERS substrates with strong magnetic properties and, due to the presence of a TiO_2_ layer, also have self-cleaning properties [51]. Shen et al. found that this material reveals some catalytic properties, for example, it may be used as a catalyst in the reduction of 4-nitrophenol to 4-aminophenol. This means that this three-layer core–shell Fe_3_O_4_@TiO_2_@Ag-Au composite shows great potential as a multifunctional platform for simultaneous catalysis and in-situ reaction monitoring.

Another material that, in addition to the possibility of generating strong SERS signal, can be used for photocatalysis and photovoltaic applications are anatase TiO_2_ nanospheres covered with gold nanoparticles [52]. Damato et al. synthetized such a system attaching gold nanoparticles to well-defined (with a diameter of ca. 230 nm) TiO_2_ nanospheres that have been obtained by the hydrolysis of spheres formed from titanium glycolate. 

## 4. SERS Substrates That Do Not Contain Nanostructured Metals

As mentioned in the introduction, around illuminated nanoobjects formed from plasmonic metals (e.g., Au, Ag or Cu), a very large enhancement of the intensity of the electric field may be observed, which leads to very large increase in the efficiency of the generation of Raman signal by molecules placed in such places. Therefore, typically used SERS substrates contain nanostructured plasmonic metals, and, for such substrates, the total SERS enhancement is dominated by the electromagnetic part. There are also, however, SERS substrates that do not contain any metallic parts, and, in the case of SERS measurements carried out on such substrates, the SERS enhancement is often dominated by the chemical mechanism. Below some typical SERS substrates that do not contain nanostructured metals are presented. As such substrates are often used to study the interaction with the surface of the material from which the substrate is produced, a large number of various materials were nanostructured to record SERS spectrum. Below we present selected examples of SERS-active materials that we consider as the most interesting. 

### 4.1. Titanium(IV) Oxide

Titanium(IV) oxide was the first non-metallic material on which SERS spectrum was observed. In 1982, Loo presented the SERS spectrum of I_2_ adsorbed on the surface of polycrystalline TiO_2_ [8]. In this case, polycrystalline TiO_2_ was prepared by heating a titanium foil in a Bunsen burner to redness for about 1 min. TiO_2_ seems to be very important material from which SERS substrates are formed, and therefore, the presentation of non-metallic SERS substrates is started from substrates made from TiO_2_. 

**Figure 11:**
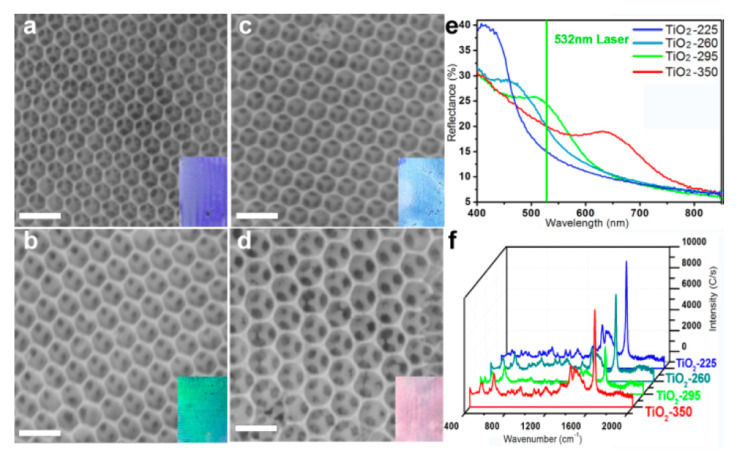
(**a**–**d**) SEM images of TiO_2_ inverse opal substrates calcined in various temperatures: (**a**) 225, (**b**) 260, (**c**) 295, (**d**) 350 °C. (**e**) Reflection spectra of TiO_2_ inverse opal substrates. (**f**) SERS spectra of methylene blue adsorbed from 10^−^^5^ M aqueous solution on various inverse opal substrates. Reprinted with permission from [53]. Copyright 2014 American Chemical Society.

Interesting SERS substrates formed from TiO_2_ have been produced by Qi et al. [53]. At first, this group deposited on a glass slide an agglomerate of polystyrene nanospheres and evaporated the solvent. Then, to the agglomerate of polystyrene nanospheres, the solution of titanium isopropoxide was applied by spincoating. The prepared substrate was dried at 75 °C for 24 h and then heated at the temperature of 450 °C for 2 h. In this way, polystyrene particles were removed and the sponge anatase structure of TiO_2_ was formed, which was described by the authors as an inverse opal photonic microarray (see Figure 11). The band gap of this material depends on the size of the pores, and their size depends on the particle size of the polystyrene nanospheres used. It was noticed that if the photonic band gap corresponds to the energy of the excitation photons used for the SERS measurements, the recorded SERS spectra are the most enhanced (the achieved SERS enhancement factor was estimated as equal to 2 × 10^4^) [53]. In addition, this SERS TiO_2_ substrate, similar to many other anatase substrates, has self-cleaning properties under the influence of the sunlight, for example, this opal TiO_2_ substrate can decompose adsorbed methylene blue after 10 min irradiation with solar light [53].

Yang et al. investigated how the crystallographic structure of the TiO_2_ nanoparticles (anatase, rutile or a mixture of both phases) influences the SERS enhancement factor generated by such systems [54]. At first, they synthesized TiO_2_ nanoparticles by the sol-hydrothermal method. Briefly, tetrabutyl titanate dissolved in anhydrous ethanol was added to the mixture of ethanol, water, and nitric acid. Subsequently, the obtained yellowish transparent sol was kept at 160 °C for 6 h in a stainless-steel vessel. Finally, the product was calcined at six different temperatures. The temperature of calcination affects the crystal phase of the obtained TiO_2_ nanoparticles. In the case of low calcination temperature (400 and 450 °C), the synthesized nanoparticles exhibit pure anatase phase. The samples calcined in high temperature (650 °C) were completely converted into rutile phase. The product calcined between 450 and 650 °C contains both phases. Yang et al. found that the TiO_2_ nanoparticles with various phase compositions generate various SERS enhancement factors. In the case of the measurement of the SERS spectra of 4-mercaptobenzoic acid adsorbed on the obtained TiO_2_ nanoparticles, the highest Raman signal is observed when the ratio of the anatase and rutile phase is approximately 85% and 15%. The TiO_2_ nanoparticles containing both crystal phases generate larger chemical SERS enhancement probably because the separation of the photogenic charge carriers is easier in the mixture of two phases [54].

In addition to the influence of the phase composition of the TiO_2_ nanoparticles on their SERS activity, defects introduced to the crystal structure of the TiO_2_ nanoparticles (by doping) may also influence the generated SERS enhancement factor. Xue et al. synthetized TiO_2_ and Mn-doped (1%, 3% and 5%) TiO_2_ nanoparticles by a sol-hydrothermal method [55]. Obtained nanoparticles have been tested as substrates for SERS measurements using 4-mercaptobenzoic acid as a Raman scatterer. Xue et al. found that doping of the nanoparticles increases the intensity of the measured Raman signal—the highest SERS signals (by a factor of six higher than the SERS signal measured on pure TiO_2_ nanoparticles) were observed on the Mn–TiO_2_ (3%) samples. Xue et al. suggested that an appropriate amount of Mn dopant enriches the surface states and improves the efficiency of photo-generated carrier separation, which leads to the increase in the efficiency of the TiO_2_-to-molecule charge-transfer process and hence, enhances the SERS intensity due to the increase in the chemical enhancement [55]. A similar effect was observed for TiO_2_ nanoparticles doped with Zn [56]. The obtained material exhibited the same crystal structure as TiO_2_ nanoparticles doped with Mn. Similar to TiO_2_ Mn-doped nanoparticles, the highest SERS signal is observed for Zn loading equal to ca. 3%. 

### 4.2. Silver Halides

**Figure 12:**
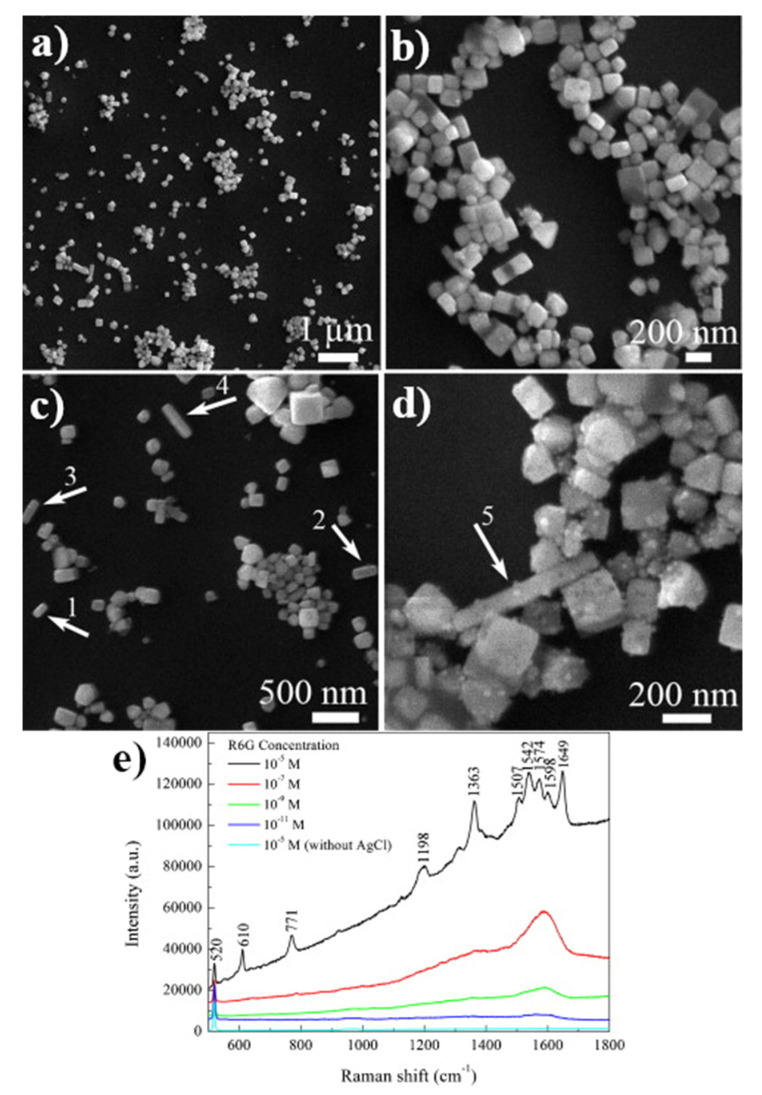
(**a**–**d**) SEM images of the AgCl nanoparticles produced by laser ablation, (**e**) SERS spectra of molecules of rhodamine 6G on AgCl nanoparticles adsorbed from solutions with various concentrations. Reprinted with permission from [64]. Copyright 2012 Elsevier.

The second (after TiO_2_) non-metallic substrate used for SERS experiments (already in 1984) was a sol of silver chloride [57]. Slightly later (in 1986), the same group showed that also a colloid of silver bromide may be used as a SERS substrate [58]. For these substrates, the enhancement of the recorded SERS spectra was not high, typically one order of magnitude. Although a very simple method of preparation of SERS-active colloids of silver halides; typically, such colloids are formed just by mixing of silver salt with halide salt, after an initial significant interest in this type of SERS substrate, for example see: AgCl [59,60], AgBr [58,61,62] or AgI [63], this type of SERS substrate is not currently in wide use. Some interesting SERS substrates made from silver halides are, however, still being developed. For example, in 2012, Dang and co-workers synthesized SERS-active cubic AgCl nanocrystals by a laser ablation—see Figure 12 [64]. Recently (in 2019), Joo et al. synthesized SERS-active highly monodisperse AgCl nanospheres and nanocubes using a rapid one-pot room-temperature aqueous synthesis [65]. 

### 4.3. Copper Oxides

An important group of SERS substrates that do not contain nanostructured metals are nanoparticles of copper oxides. The first report concerning SERS activity of nanostructured Cu_2_O was published in 1998 by Kudelski et al. [66] (it was the second reported in the literature of SERS-active oxide of a metal that is standardly used to create SERS substrates, the first kind of this type of SERS substrates was colloidal silver oxide [67]). Various Cu_2_O microcrystals have been tested as SERS substrates by Lin et al., who synthesized highly uniform cubic, rhombic dodecahedral, and octahedral Cu_2_O microcrystals with well-defined {100}, {110}, and {111} facets and clear surface atomic configuration. Cu_2_O {100}-cubic nanoparticles were synthesized by the reduction of the alkalized (by the addition of NaOH) solution of CuCl_2_ by the solution of ascorbic acid. Then, {111}-octahedral Cu_2_O nanoparticles were obtained using similar procedure; however, a significant amount of polyvinylpyrrolidone was added to the solution of CuCl_2_. Then, {110}-dodecahedral Cu_2_O nanoparticles were synthesized by the reduction by D-(+)-glucose of the solution obtained by mixing solutions of CuSO_4_ and NaOH and oleic acid and ethanol. As one can expect, the interaction of adsorbed molecules with surfaces of the adsorbent with different orientations leads to various complexes, for which different SERS enhancement factors are observed [68]. Lin et al. found that the ratio of SERS enhancement factors for 4-nitrobenzenethiol adsorbed on {100}, {110}, and {111} facets of Cu_2_O microcrystals is about 15:6:2 [68]. These clearly demonstrate that regarding the SERS measurements on semiconductor substrates, choosing nanoobjects with the proper crystallographic orientation of the outermost surfaces is very important.

It is well known that aggregates (superstructures) formed from plasmonic nanoparticles generate significantly stronger SERS signals than isolated nanoparticles [5]. This is due to the coupling of plasmons induced in the individual plasmonic nanostructures, which generate a very strong electric field in slits between nanoparticles [5]. Lin et al. found a similar effect in clusters of Cu_2_O nanoparticles, a significantly increased SERS enhancement factor in comparison to the individual nanoparticles, which was explained by the resonance coupling between charge-transfer complexes formed from Cu_2_O and the adsorbed molecules. Cu_2_O superstructures studied Lin et al. were cube-like, with a size of about 2 μm and were formed from Cu_2_O mesoporous spheres with a diameter of ca. 300 nm. SERS enhancement factor generated by these super-structures of Cu_2_O nanoparticles was comparative to the enhancement factor generated by standard plasmonic SERS substrates and was estimated as 8 × 10^5^ [69].

### 4.4. Molybdenum Oxides

**Figure 13:**
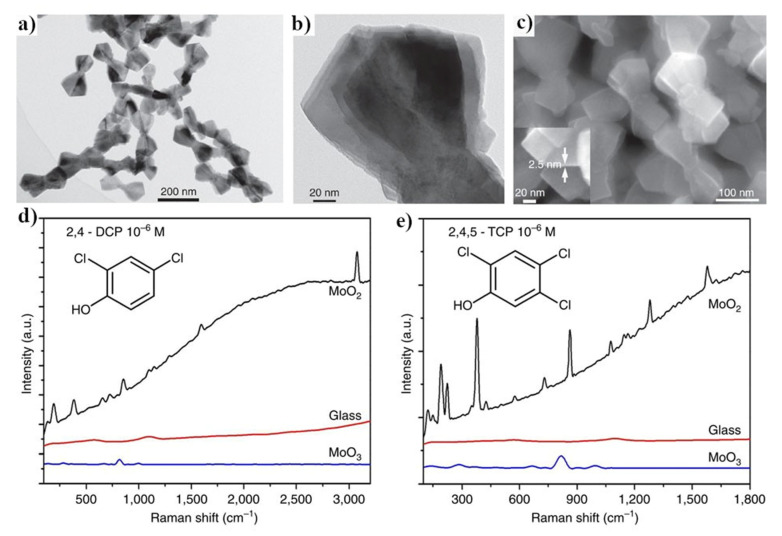
(**a**,**b**) TEM images of the MoO_2_ nanocrystals, (**c**) SEM image of the MoO_2_ nanocrystals, (**d**,**e**) SERS spectra of polychlorinated phenols adsorbed on the surface of MoO_2_. Reprinted with permission from [70].

Very large SERS enhancement factors (even reaching the values generated by standard silver and gold SERS substrates) may be also obtained on nanostructured molybdenum dioxide. For example, Zhang et al. synthesized MoO_2_ nanoparticles by a simple hydrothermal method, briefly: water and ethanol solution of molybdenyl acetylacetonate was kept (in autoclave) at 180 °C for 20 h—see Figure 13 [70]. Formed MoO_2_ nanoparticles exhibit plasmonic properties. Obtained material is very stable chemically; the plasmonic band does not change neither its shape nor the position even after contact with many corrosive substances or after heating in air at 300 °C for 24 h. The SERS enhancement factor generated by this substrate was estimated as equal to 3.75 × 10^6^. In the next contribution, this group slightly modified the experimental conditions of the synthesis of MoO_2_ nanoparticles (isopropanol was added to the reaction mixture), obtaining significantly more regular nanoparticles with the average diameter of about 40 nm, which generated a slightly higher SERS enhancement factor (ca. 4.8 × 10^6^) [71].

Chen et al. attached MoO_2_ nanoparticles to the surface of graphene oxide by a simple hydrothermal procedure [72]. This allowed the obtention of a very active SERS material, which generates a SERS enhancement factor estimated as equal to ca. 1.05 × 10^7^. Formed SERS substrate exhibits very good time stability, the SERS signal decreases only by 4.4% after 40 days. Additionally, in the case of experiments with some organic dyes, graphene oxide significantly reduced in the recorded SERS spectra of the fluorescence background usually generated by molecules of dyes.

**Figure 14:**
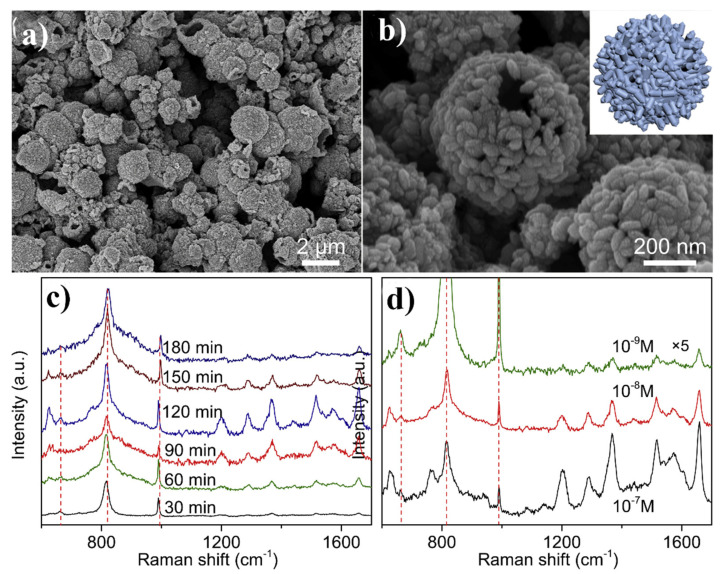
(**a**,**b**) SEM images of hollow MoO_2_ spheres, inset shows the schematic structure of this nanomaterial, (**c**) SERS spectra of rhodamine 6G adsorbed on the hollow MoO_2_ nanospheres after various anodization times from 10^–7^ M rhodamine 6G solution, (**d**) SERS spectra of rhodamine 6G adsorbed from solutions with different concentrations on the hollow MoO_2_ nanospheres. Reprinted with permission from [73].

Further increase in the SERS enhancement factor generated by MoO_2_ nanoparticles was obtained by the formation of hollow MoO_2_ nanostructures. Zhang et al. synthesized hollow MoO_2_ spheres by the self-assembly of MoO_2_ nanocones—see Figure 14 [73]. The initial MoO_2_ nanocones (with a length in the range of 40 and 100 nm and a diameter of ca. 20 nm) were synthesized by anodizing the molybdenum bar in methanol and toluene in the presence of tetramethylammonium bromide as surfactant. Then, the sol of MoO_2_ nanocones was kept in an autoclave at 260 °C for 24 h, which lead to the arrangement of MoO_2_ nanocones in spheres (with the size in the range of 500 nm—3 μm). Zhang et al. determined the SERS enhancement factor generated by this system as equal to 3 × 10^7^ [73].

Additionally, another molybdenum oxide (MoO_3_) after proper nanostructuring may be used as SERS substrate. Prabhu et al. deposited on glass or silicon MoO_3_ nanostructures with a morphology of sea urchin with ca. 15 μm long spikes (with taper-shape 20 nm sharp tips) originating from a 20–40 micron globular core [74]. The formation of MoO_3_ was performed employing a simple chemical bath deposition method, briefly: the cleaned glass or silicon substrates were dipped for 3 h in an aqueous solution of (NH_4_)_6_Mo_7_O_24_·4H_2_O and HNO_3_ heated to 90 °C. The SERS enhancement factor generated by this substrate was estimated as equal to ca. 10^7^ [74]. Zhang et al. have also shown that it is possible to use molybdenum oxides with tunable phases as SERS substrates (MoO_2_, MoO_3__−__x_, and MoO_3_) [75,76].

### 4.5. Zinc Oxide

Many various SERS substrates have been formed from zinc oxide or doped zinc oxide. A simple method of synthesis of SERS-active ZnO nanocrystals was proposed by Wang et al. [77], briefly: a reaction mixture obtained by mixing aqueous solutions of NaOH and ZnCl_2_ was stirred for 2 h at 90 °C. The size of formed ZnO nanocrystals was estimated as equal to 20 nm. When 4-mercaptopyridine is used as a Raman scatterer, the SERS enhancement factor generated by this material (most likely due to the chemical enhancement) was estimated as equal to 10^3^ [77]. Formation of some more complex structures from ZnO allows the significant increase in the generated SERS enhancement factor. For example, for the same Raman scatterer (4-mercaptopyridine), the hollow amorphous ZnO nanocages with a size of about 600 nm generate a SERS enhancement factor equal to 6.62 × 10^5^ [78]. ZnO nanocages were synthesized using Cu_2_O nanocubes as a template.

A proper doping of ZnO nanocrystals can significantly increase their SERS activity. For example, Xue et al. observed an increase in the SERS activity of ZnO nanoparticles after doping with Co [79]. Recently, Gao et al. fabricated the neodymium-doped zinc oxide nanoparticles (the Zn:Nd ratio in the formed crystals was 98:2) and showed that such nanoparticles generated a seven times higher SERS enhancement factor than that generated by nanoparticles of pure zinc oxide [80]. As in the described above cases, 4-mercaptopyridine was chosen as a probe molecule. For ZnO substrates, the SERS enhancement is primarily generated by the chemical affect due to the charge-transfer between the adsorbed molecules and semiconductor. Doping generates more defects that can act as charge carrier recombination sites. The synthesis of neodymium-doped zinc oxide nanoparticles was very simple, in brief: to the aqueous solution of Zn(NO_3_)_2_ and Nd(NO_3_)_3_, the aqueous solution of NH_4_HCO_3_ was added, a white precipitate—formed immediately after adding NH_4_HCO_3_—was washed, dried at 80 °C and further annealed in air at 600 °C. 

SERS-active materials may be also obtained by the deposition of ZnO nanoparticles on other nanostructured substrates. Quan et al. synthesized such substrates by the deposition of ZnO nanoparticles on MoS_2_ microflowers [81]. Analysis of the obtained material showed that the ZnO nanoparticles with sizes in the range from 16 to 25 nm were homogenously distributed on the surface of flower-like MoS_2_ microstructures. The SERS enhancement factor generated by this system was estimated as equal to 5.8 × 10^5^ [81].

### 4.6. Examples of Other Materials

Another very promising material from which SERS substrates can be generated is niobium(V) oxide. In 2017, Shan et al. showed that Nb_2_O_5_ nanoparticles can generate very large SERS enhancement factors (similar to those generated by standard silver or gold SERS substrates), for example, in SERS experiments using excitation radiation with the wavelength of 532, 633 and 780 nm, the SERS enhancement factor was determined as equal to: 2.6 × 10^6^, 1.9 × 10^7^ and 7.1 × 10^7^, respectively [82]. Moreover, SERS substrates generated from Nb_2_O_5_ nanoparticles exhibit very good reproducibility, for example, the measured intensity of eighteen Raman spectra recorded on six various Nb_2_O_5_ samples in three different spots showed relative standard deviation smaller than 10%. 

Large SERS enhancement factors were also measured in SERS experiments on Zn-doped ZrO_2_ nanoparticles [83]. Such nanoparticles were formed by the addition of triemethylamine to the aqueous solution of zirconium nitrate and zinc nitrate. Separated product was dried and washed by ethanol. Finally, the product was calcinated in 500 °C for 2 h. It was found from the XPS analysis that Zn doping decreases the band gap from 4.95 eV in the case of pure ZrO_2_ nanoparticles to even 3.08 eV for 5% Zn doping. This is probably due to the increasing number of defects with the increasing amount of Zn ions. The highest SERS activity exhibited ZrO_2_ nanoparticles with 1% Zn doping. In SERS experiments with p-mercaptobenzoic acid as a Raman reporter, the SERS enhancement factor was determined as equal to 1.94 × 10^4^ [83]. 

In addition to the listed above non-metallic SERS-active materials, which we chose to describe in more detail, the SERS substrates have been also generated from many other compounds, for example (the estimated SERS enhancement factor is given in brackets): V_2_O_5_ [84], GaP (700) [85], ZnS (10^3^) [86], CdS (10^3^) [87], CdSe (2 × 10^3^) [88,89], PbS (800) [90], graphene (3.2 × 10^3^) [91] (2370) [92], InAs/GaAs (10^3^) [93], WO_3−x_ [94], PbTe [95], boron-doped diamond (BDD) (10^4^–10^5^) [96], SnO_2_ (10^3^) [97], WS_2_ [98], MoS_2_ (10^5^) [98], TiN (3.5 × 10^3^) [99,100] or CdTe (10^4^) [101]. The detailed description of all SERS-active materials, as in the case of the described above non-metallic nanostructured thin films or non-metallic nanoparticles covered by plasmonic metals, is, however, beyond the purpose of this work. 

## 5. Conclusions

SERS spectroscopy is one of the most sensitive analytical tools. In some cases, the generated SERS signal is so strong that it is possible to record a reliable SERS spectrum even of a single molecule. Therefore, SERS experiments are carried out by a very large number of research groups. To obtain a SERS spectrum, the studied molecules must be adsorbed on a SERS-active substrate (or placed at a very close distance to such a substrate). The quality of measured SERS spectra is closely correlated with the activity of used SERS substrates. In this review article, selected SERS substrates that utilize nanostructured non-metallic materials are described. Recently, one can observe a significant progress in the construction of such a type of nanomaterials. Obtained materials, even those that do not contain plasmonic metals, can generate very large SERS enhancement factors—in some cases similar to those generated on standard SERS substrates produced from pure gold or silver. Moreover, SERS substrates that utilize nanostructured non-metallic materials are often significantly more reproducible than the standard metallic SERS substrates. They can also have some additional functionalities, for example, they can very effectively self-clean when illuminated with UV radiation or can be manipulated by a magnetic field (in the case of magnetic–plasmonic composites), which significantly facilitates their homogenous deposition and removal after the measurement. Therefore, we think that SERS substrates that utilize nanostructured non-metallic materials are a very promising group of nanomaterials and we expect a significant continuous development in this field in the near future—especially in the development of multifunctional SERS substrates, and those designed for specific applications. The strengths and weaknesses of different types of SERS substrates formed from nanostructured non-metallic materials are listed in Table 2.

## Figures and Tables

**Table 1:** The correlation between the amount of silver deposited by sputtering on the layer of TO_2_ nanotubes and the structure of the formed metallic layer.

Amount of Silver Sputtered [mg∙cm^−^^2^]	Type of Silver Nanostructures
0.01	nanoparticles from 10 to 50 nm in diameter
0.06	nanoparticles from 10 to 50 nm in diameter
0.09	ring on top of nanotubes, reduced light of the tubes, visible nanoparticles with a diameter of 10 to 50 nm on the tube walls
2	complete surface coverage, no nanotubes can be seen

**Table 2:** Comparison of different types of SERS substrates formed from nanostructured non-metallic materials.

Material	Strengths	Weaknesses
non-metallic nanostructured thin films covered by plasmonic metals	usually high reproducibility, usually high SERS enhancement factors	domination of the measured spectrum by the contribution from molecules interacting directly with the plasmonic metal
non-metallic nanoparticles covered by plasmonic metals	usually high SERS enhancement factors, easy possibility to add additional functionalities	domination of the measured spectrum by the contribution from molecules interacting directly with the plasmonic metal
SERS substrates that do not contain nanostructured metals	measured SERS spectrum from molecules interacting directly with the analysed material	often low SERS enhancement factors

## Data Availability

All data have been illustrated in the manuscript.
